# Fabrication of Functional Bioelastomer for Food Packaging from Aronia (*Aronia melanocarpa*) Juice Processing By-Products

**DOI:** 10.3390/foods9111565

**Published:** 2020-10-28

**Authors:** Kang Hyun Lee, Youngsang Chun, Ye Won Jang, Soo Kweon Lee, Hyeong Ryeol Kim, Ju Hun Lee, Seung Wook Kim, Chulhwan Park, Hah Young Yoo

**Affiliations:** 1 Department of Biotechnology, Sangmyung University, 20, Hongjimun, 2-Gil, Jongno-Gu, Seoul 03016, Korea; oys7158@naver.com (K.H.L.); yesyewon@naver.com (Y.W.J.); 2 Department of Interdisciplinary Bio-Micro System Technology, College of Engineering, Korea University, 145 Anam-Ro 5, Seongbuk-Gu, Seoul 02841, Korea; youngsangchun@korea.ac.kr; 3 Department of Chemical and Biological Engineering, Korea University, 145 Anam-Ro, Seongbuk-Gu, Seoul 02841, Korea; sookweon@korea.ac.kr (S.K.L.); hfire003@naver.com (H.R.K.); juhunlee@korea.ac.kr (J.H.L.); 4 Department of Chemical Engineering, Kwangwoon University, 20 Kwangwoon-ro, Nowon-gu, Seoul 01897, Korea

**Keywords:** aronia, bioelastomer, biorefinery, food packaging, food waste

## Abstract

Carbon-neutral and eco-friendly biomass-based processes are recognized as a frontier technology for sustainable development. In particular, biopolymers are expected to replace petrochemical-based films that are widely used in food packaging. In this study, the fabrication conditions of functional (antioxidant and antibacterial) bioelastomers were investigated using by-products from the juice processing (experimental group) and freeze-dried whole fruit (control group). Bioelastomer was fabricated by a casting method in which polydimethylsiloxane (PDMS) was mixed with 25 or 50 wt% aronia powder (juice processing by-products and freeze-dried whole fruit). The mechanical properties of the bioelastomers were measured based on tensile strength and Young’s modulus. When the mixture contained 50 wt% aronia powder, the strength was not appropriate for the intended purpose. Next, the surface and chemical properties of the bioelastomer were analyzed; the addition of aronia powder did not significantly change these properties when compared to PDMS film (no aronia powder). However, the addition of aronia powder had a significant effect on antioxidant and antimicrobial activities and showed higher activity with 50 wt% than with 25 wt%. In particular, bioelastomers fabricated from aronia juice processing by-products exhibited approximately 1.4-fold lower and 1.5-fold higher antioxidant and antimicrobial activities, respectively, than the control group (bioelastomers fabricated from freeze-dried aronia powder).

## 1. Introduction

In recent years, petroleum-based plastics have caused greenhouse gas emissions and various environmental problems owing to the production process. Consequently, carbon-neutral and eco-friendly biopolymers have attracted great attention as alternative materials. From food sources such as corn, cassava, and sugarcane to non-food resources such as wood, agricultural and food processing by-products, sustainable biomass can be used as a raw material for biopolymer production [1,2]. The biopolymer market reached US$ 35.9 billion in 2018, 51% of which was used in the food packaging industry [3]. The global food packaging market is estimated to increase from US$ 16.1 billion in 2018 to US$ 19.6 billion in 2023, registering a compound annual growth rate (CAGR) of 3.9%, which is expected to increase the demand for biopolymers [4]. Recently, various packaging containers and durable products using bioplastics have been actively developed [5,6,7].

The fruit juice processing industry has become one of the largest markets in the agro-industrial sector [8]. Juice processing by-products are usually buried directly in the soil, which causes serious environmental pollution [9]. However, these by-products still contain bioactive compounds such as tannins, flavonoids, flavanols, vitamins, essential minerals, fatty acids, volatiles, and pigments, which can be utilized as natural bioactive compounds [10]. The scientific community has suggested the use of these natural compounds instead of synthetic compounds due to their synergy, potency, and minimal side effects [11]. These bioactive compounds have both antioxidant and antibacterial properties [12]. Antioxidants are used as food additives for the inhibition of oxidation, to increase the shelf life of foodstuffs [13]. Antibacterial agents are usually applied in edible films and coatings to improve food quality by preventing foodborne illnesses [14]. Research using bioactive compounds derived from food processing by-products to produce film-type functional materials has recently attracted attention, and related studies are summarized in Table 1. Bioactive compounds can be prepared from various food processing by-products such as grape seed, tomato skin, spinach stems, rice hulls, cocoa shell, etc., and bioelastomers are produced by blending with polymers such as polypropylene (PP), polyvinyl alcohol (PVA), low-density polyethylene (LDPE), and polydimethylsiloxane (PDMS) under various conditions. In particular, film-type functional materials can be beneficially applied in various fields such as food packaging, cosmetics, and medicine.

Aronia (*Aronia melanocarpa*) is a berry known to contain various polyphenols such as flavonols, flavanols, anthocyanins, proanthocyanidins, and phenolic acids [23]. These polyphenols have bioactive properties including antioxidant, antibacterial, antidiabetic, and anti-inflammatory activities [24]. Aronia is generally consumed as a juice rather than by direct consumption because of its tart and bitter taste [25]. After juice processing, the dry weight of aronia by-products remains at approximately 44.6 to 50% [26]. The bioactive compounds contained in the whole fruit remain in the by-products, thus, it can be used as additives for functional materials with antioxidant and antibacterial properties [27,28].

In this study, in order to utilize by-products generated from aronia juice processing as a valuable resource, functional bioelastomers that can be used for food packaging were fabricated. Since aronia juice processing by-products contain abundant natural bioactive molecules, antioxidant and antibacterial activities can be expected. In order to evaluate the potential value of the biomass, all experiments were designed by comparing the freeze-dried powder as control experiments. Based on natural bioactive compounds in aronia, bioelastomers were prepared using non-toxic and flexible PDMS as a food packaging material. In addition, the physicochemical properties of the bioelastomer fabricated according to the source and content of aronia powder were analyzed by using as follows: Fourier–transform infrared spectroscopy (FT–IR), water contact angle (CA) and field emission scanning electron microscopy (FE–SEM). Finally, the potential of produced bioelastomers as functional packaging materials was evaluated by analyzing their antioxidant and antibacterial activities.

## 2. Materials and Methods

### 2.1. Materials

Aronia was purchased from Sandlehae (Gyeongsangnam-Do, Korea), which was harvested in the Honam area, South Korea. Folin–Ciocalteu reagent, sodium carbonate (Na_2_CO_3_), gallic acid, sodium nitrite (NaNO_2_), sodium acetate trihydrate (CH_3_CO_2_Na∙3H_2_O), potassium persulfate (K_2_S_2_O_8_), 1,1-diphenyl-2-picryl-hydrazyl (DPPH), and 2,2′-azino-bis(3-ethylbenzothiazoline-6-sulphonic acid) (ABTS) were purchased from Sigma-Aldrich (St. Louis, MO, USA). Ethanol, methanol, sodium hydroxide (NaOH), and potassium chloride (KCl) were obtained from Samchun Chemical (Seoul, Korea). Aluminum chloride (AlCl_3_) was supplied by Duksan Pure Chemical (Ansan, Korea). All reagents used in the current study were of analytical grade.

### 2.2. Preparation of Bioelastomers

Aronia juice was extracted at 23,000 rpm using a blender (WellbeingQ, Gapo, Seoul, Korea), and solid residues (by-products of aronia juice processing) were collected and dried in an oven at 40 °C for three days. In addition, the whole fruit of aronia was dried in a freeze-dryer, and the freeze-dried powder was used as a raw material for control experiments. Each powder was sieved to a diameter of 90 µm or less using a test sieve (Ø203 × 41 mm, Chung Gye Sang Gong Sa, Seoul, Korea). Aronia powder was added to 10 mL of heptane and mixed vigorously in a conical tube for 10 min to obtain a homogeneous suspension. PDMS (Elastosil E43, Wacker, Munich, Germany) was mixed with the aronia-heptane mixture. The suspension was poured into a Petri dish and dried at room temperature to form the film-type of a bioelastomer. Table 2 presents the detailed conditions of the powder and PDMS composition for aronia bioelastomer formation.

### 2.3. Characterization of Bioelastomers

FT–IR (Frontier, Perkin Elmer, Waltham, MA, USA) was used to analyze chemical properties of the powders (freeze-dried aronia and aronia juice processing by-products powder) and films (PDMS, control group, and experimental group). Both the control and experimental groups were made as a disk-type with potassium bromide, and the bioelastomers were confirmed by attenuated total reflectance (ATR)-mode. The water CA was measured using CA measurements (phx300, SEO, Suwon, Korea) with a droplet of distilled water on the surface of the prepared bioelastomer. The morphology of the bioelastomers was investigated using FE–SEM (S-4800, Hitachi, Tokyo, Japan) at 15 kV. The samples were prepared by sputter coating with a thin Pt layer on a double-adhesive carbon disk to avoid charging problems. The mechanical properties of the bioelastomers were evaluated using a universal testing machine (UTM; model 4467, Instron, Norwood, MA, USA) following American Society for Testing and Materials (ASTM) D882. All experiments were performed in triplicate to indicate standard deviation.

### 2.4. Antioxidant Activity of Bioelastomers

#### 2.4.1. Total Polyphenol Content

To extract antioxidants from bioelastomers, 2 g of bioelastomer was soaked in 20 mL 50% ethanol at 100 °C for 1 h. Total polyphenol content was measured by modifying the Folin–Ciocalteu colorimetric method [29]. Then, 10 μL of the extract was mixed with 790 μL distilled water and 50 μL Folin–Ciocalteu reagent and reacted at 30 °C for 8 min. Subsequently, 150 μL of 20% sodium carbonate solution was added to the mixture, followed by reaction at 25 °C for 1 h. Optical absorbance was measured at 765 nm using a spectrophotometer (DU 730, Beckman Coulter, Brea, CA, USA). Total polyphenol content was expressed as mg gallic acid equivalent (GAE) per g of dry powder (mg GAE/g dry powder). All experiments were performed in triplicate to indicate standard deviation.

#### 2.4.2. Total Flavonoid Content

To extract antioxidants from bioelastomers, 2 g of bioelastomer was soaked in 20 mL 50% ethanol at 100 °C for 1 h. The aluminum chloride colorimetric method was used to quantify total flavonoid content [30]. A total of 30 μL of 5% NaNO_2_ solution was added to 50 μL of extract and reacted at 25 °C for 6 min. Subsequently, 50 μL of 10% AlCl_3_ solution was added to the mixture and reacted at 25 °C for 5 min. Finally, 300 μL of 1M NaOH and 1000 μL of distilled water were added and reacted at 25 °C for 15 min. Optical absorbance was measured at 510 nm. Total flavonoid content was expressed as mg rutin equivalent (RE) per g of dry powder (mg RE/g dry powder). All experiments were performed in triplicate to indicate standard deviation.

#### 2.4.3. DPPH Radical Scavenging Activity

To extract antioxidants from bioelastomers, 2 g of bioelastomer was soaked in 20 mL 50% ethanol at 100 °C for 1 h. DPPH radical scavenging activity was analyzed to determine antioxidant activity [31]. First, 500 μL of the extract was reacted with 500 μL of 0.5 mM DPPH solution at 25 °C for 30 min; then, absorbance was measured at 517 nm. The blank was 1 mL methanol, and the control was a mixture of 500 μL methanol and 500 μL 0.5 mM DPPH solution. DPPH radical scavenging activity was calculated according to the following equation:

DPPH radical scavenging activity (%) = (1 − (OD _sample_/OD _control_)) × 100 (1)

All experiments were performed in triplicate to indicate standard deviation.

#### 2.4.4. ABTS Radical Scavenging Activity

To extract antioxidants from bioelastomers, 2 g of bioelastomer was soaked in 20 mL 50% ethanol at 100 °C for 1 h. An ABTS radical cation decolorization assay was used to estimate antioxidant activity [32]. ABTS^•+^ cation radical was reacted with 7 mM ABTS solution and 2.45 mM potassium persulfate (1:1) for 12 h before use and stored at room temperature. ABTS^•+^ solution was diluted with methanol until the absorbance at 734 nm reached 0.7. A total of 950 μL of diluted ABTS^•+^ solution and 50 μL of extract were added and reacted at 25 °C for 30 min, and then absorbance was measured at 734 nm. The blank was 1 mL of methanol and the control was a mixture of 950 μL of diluted ABTS^•+^ solution and 50 μL of methanol. ABTS^•+^ radical scavenging activity was calculated according to the following equation:

ABTS radical scavenging activity (%) = (1 − (OD _sample_/OD _control_)) × 100 (2)

All experiments were performed in triplicate to indicate standard deviation.

### 2.5. Antibacterial Activity of Bioelastomers

Bioelastomer antibacterial activity was evaluated using *Staphylococcus aureus* as Gram-positive bacteria and *Escherichia coli* as Gram-negative bacteria. Bacteria were cultured in nutrient broth (NB) at 37 °C for 24 h with 150 rpm rotary agitation. The cultured bacteria were diluted to 10^6^ colony forming unit (CFU)/mL and inoculated onto nutrient agar plates. Bioelastomers were cut to a size of 1 × 1 cm and placed on each agar plate, which were then incubated at 37 °C for 24 h. All experiments were performed in triplicate to indicate standard deviation. The characteristics of antibacterial activity were determined by the area of inhibition, which was calculated using Image J software (v1.52i, National Institutes of Health, Bethesda, MN, USA).

## 3. Results and Discussions

### 3.1. Mechanical Properties of Bioelastomers

**Figure 1:**
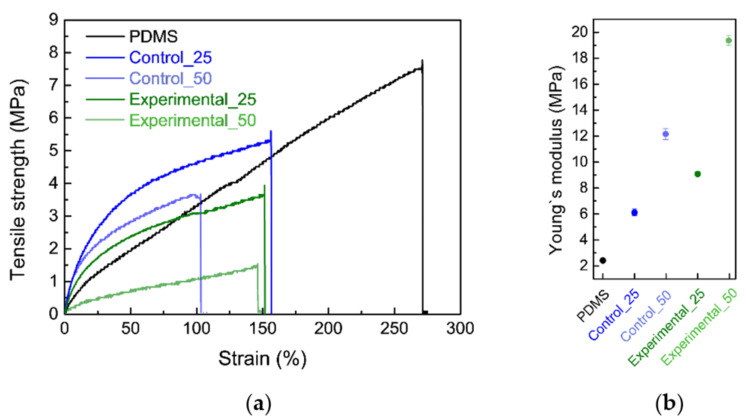
Tensile stress-strain curves (**a**) and Young’s modulus (**b**) of different bioelastomers (PDMS, Control_25, Control_50, Experimental_25, and Experimental_50).

The mechanical properties of the bioelastomers were examined for their stable use in applications such as packaging (Figure 1). Both tensile strength and Young’s modulus were significantly influenced by the addition ratio of aronia powders in the bioelastomers. The addition of powders reduced the tensile strength of the bioelastomers due to the low physicochemical interaction of the silicon network, thus causing the bioelastomers to become brittle (Figure 1a) [19]. Young’s modulus of Control_50 and Experimental_50 was higher than that of PDMS, Control_25, and Experimental_25. High loading of powders negatively affected the interactions of the silicone network, causing loss of elastomeric properties. The relatively stiff bioelastomers displayed low elongation levels before breakage owing to a low degree of cross-linking with the heterogeneous constitutions of by-products. Furthermore, all bioelastomers had Young’s modulus higher than 2.42 ± 0.04 MPa of PDMS because of the random distribution of powders in the composite which could change the extent of PDMS cross-linking (Figure 1b) [33]. A similar trend in the reduction of Young’s modulus was observed in previous studies of starch-based bioelastomers containing red beetroot and PDMS bioelastomers containing cocoa shell waste [13,19]. In addition, in bioelastomers prepared using >50 wt% powder, the PDMS framework was easily broken, which rendered them unsuitable for use as the final products. An appropriate amount of powder should be considered before manufacturing bioelastomers.

### 3.2. Surface Properties of Bioelastomers

**Figure 2:**
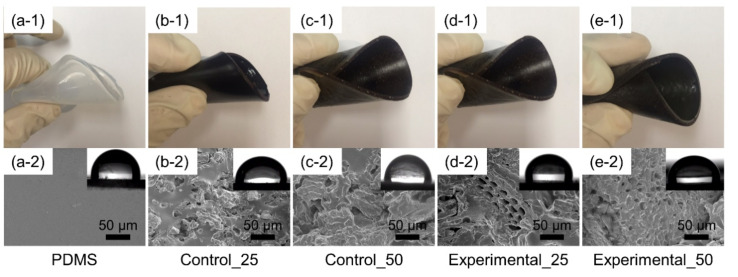
Photographs (upper section denoted by 1) of the rolling state of the bioelastomers. Field emission scanning electron microscopy (FE-SEM) images (lower section denoted by 2) of bioelastomer surfaces. Inset shows the water contact angle of the bioelastomers. (a: PDMS, b: Control_25, c: Control_50, d: Experimental_25, and e: Experimental_50).

**Figure 3:**
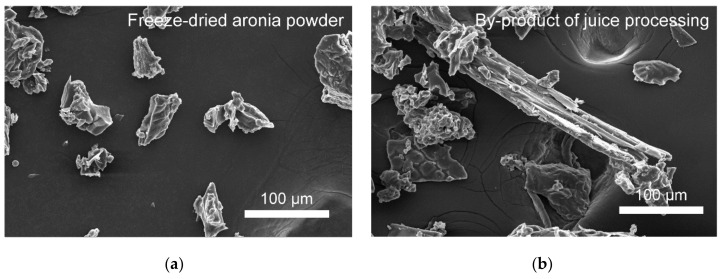
FE-SEM images of aronia freeze-dried powder (**a**) and aronia juice processing by-products (**b**).

The film-type of a bioelastomer of PDMS-based composites can be deformed and remain flexible, as shown in the upper section of Figure 2. Transparent PDMS (Figure 2(a-1)) became dark brown upon addition of aronia powder (control and experimental groups; (Figure 2(b-1, c-1, d-1, e-1)). The different macroscopic surfaces of aronia power-added bioelastomers were investigated (Figure 2 (lower section)). The surface morphology of PDMS was smooth, as shown in Figure 2(a-2). With an increase in the powder content of the bioelastomers (25 wt% and 50 wt%), agglomerations of powders within the PDMS matrix appeared rougher and more porous. As shown in Figure 2(d-2, e-2), large pores were observed on the surface of the bioelastomers owing to the by-products, namely, berries, seeds, and remaining branches. As shown in Figure 3, powder type was observed in the control group; however, the experimental group consisted of powders and long branches, which may affect the surface morphology of the bioelastomers. We expected that the porous surfaces of Experimental_25 and Experimental_50 were related to the shape and compositions of the simulated aronia processing by-products (Experimental group). The water CA was measured to characterize the surface chemical properties of the bioelastomers. As shown in Figure 2(a-2) (inset), water CA was almost ≈90° due to the hydrophobic property of PDMS that is attributed to the presence of methyl groups (Si–CH_3_). Static water CA was also affected by the surface roughness of all the bioelastomers contributing to hydrophobic properties. The water CA of the bioelastomers was similar to that of PDMS; no significant changes in water CA were observed (Figure 2(a-2,b-2,c-2,d-2,e-2) (inset)). The rough surface and chemical properties of the bioelastomers also contributed to high water CA (≥90°); however, the porous structure of the bioelastomers was expected to increase mass transfer properties [34].

### 3.3. Chemical Properties of Aronia Powders and Bioelastomers

**Figure 4:**
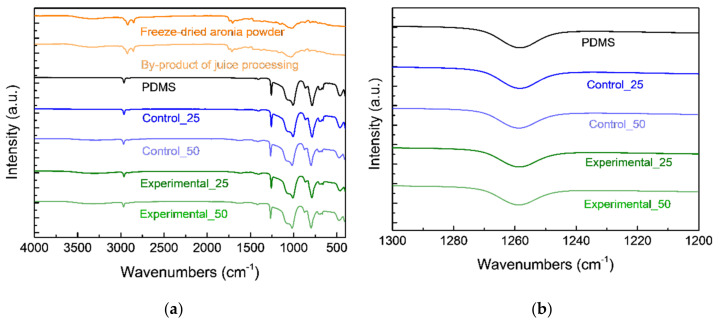
(**a**) Attenuated total reflectance–Fourier transform infrared spectroscopy (ATR-FTIR) spectra of aronia freeze-dried powder, aronia juice processing by-products, PDMS (0 wt%), Control_25, Control_50, Experimental_25, and Experimental_50; (**b**) details of Si–CH_3_ bands of bioelastomers.

In the FT–IR spectrum for powders (control and experimental groups), a broad band at around 3400 cm^−1^ indicating O–H stretching vibration from polysaccharides, and two sharp peaks of C–H asymmetric/symmetric stretching vibration at 2920 cm^−1^ and 2850 cm^−1^ from the lipid content appeared, as shown in Figure 4a [35]. The absorption peak of the carbonyl C=O stretching at 1740 cm^−1^ was assumed to be due to the presence of polyphenol and flavonoid compounds such as rutin. The spectrum of PDMS presented peaks of CH_3_ asymmetric/symmetric stretching at 2965 cm^−1^ and 2905 cm^−1^, respectively [36].

The acetoxy peak of PDMS was not evident owing to the release of acetic acid during long curing times [19]. Other strong bands assigned to Si–O–Si asymmetric/symmetric stretching were observed at 1065 cm^−1^ and 1010 cm^−1^. In addition, the methyl symmetric bending of Si–CH_3_ showed strong intensity at 1260 cm^−1^ [13]. These peaks indicated the polymerization of PDMS due to the addition of powders. After the blending of PDMS and powders, the strong peak of Si–O–Si shifted to a slightly higher wavenumber as the amount of powder added was increased (Figure 4b). We regarded that changes in the vibrational energy of the siloxane, or the electronegativity of the Si atom, were due to alterations involving the components [37]. The degree of PDMS curing should be examined to utilize the effective performance of the silicone polymer prior to use. Although biomass powder was mixed to form a bioelastomer, a curing process occurred through a hydrolysis reaction between hydroxyl-terminated PDMS and triacetoxymethylsilane cross-linker under conditions of mild moisture.

### 3.4. Antioxidant Activity of Aronia Powders and Bioelastomers

The polyphenol and flavonoid contents in extracts of two different powders and the bioelastomer are shown in Table 3. The content of phenolic compounds can be used as a significant indicator of antioxidant activity and can be applied to select food packaging materials [38]. The results showed that the experimental group contained 80% polyphenols and 90% flavonoids in comparison to the control group. After harsh juice processing, the experimental group still presented 37.2 ± 1.3 mg GAE/g dry powder polyphenols, and 18.5 ± 0.8 mg RE/g dry powder flavonoids, which indicated that aronia processing by-products are an effective resource for antioxidant materials. Various studies have also reported that phenolic compounds remain in by-products after juice processing [9,39,40,41]. However, the polyphenols and flavonoids extracted from the bioelastomer were found to be lower than the powder. It was difficult for the ethanol/water solution to gain access to the inside of the PDMS, with water CA ≈ 90°, indicating hydrophobic surface properties. In the bioelastomers, the contents were shown to be exactly proportional to the amount of powder.

Flavonoids exert a potent antioxidant effect by removing free radicals through metal ion chelation, and by providing electrons or hydrogen atoms [42]. Anthocyanins belong to a subclass of flavonoids and remove free radicals, preventing the oxidation of cells [43]. Oxidation causes food decomposition, involving lipids, nutrients, and pigment destruction, so adding antioxidants to food packaging materials can extend the shelf life and improve quality [44]. Aronia has been reported to be rich in anthocyanins such as cyanidin-3-O-glucoside, cyanidin-3-O-xyloside, cyanidin-3-O-galactoside, and cyanidin-3-O-arabinoside [45]. Flavonoid glycosides are a form of sugar bound to flavonoids, which improve the physicochemical properties of hydrophobic flavonoids, including solubility, intra- and intercellular transport, chemical stability, and biological half-life [46]. Flavonoid glycosides, which are known to have higher antioxidant activity than flavonoid aglycone in vitro, are considered to be more suitable as food packaging materials [47].

DPPH and ABTS assays were performed to measure the radical scavenging activity of the bioelastomers, as shown in Table 4. Measuring DPPH and ABTS radical scavenging activities are the most common and simple methods to determine the antioxidant activity of hydrophilic and lipophilic substances in food science [48]. Aronia freeze-dried powder showed 1.4-fold and 2.1-fold stronger radical scavenging activity than aronia juice processing by-products in DPPH and ABTS assays, respectively. The control groups showed approximately 1.3 to 1.6-fold higher radical scavenging activity than the experimental groups. These results indicated that Experimental_25 and Experimental_50 still had effective antioxidant activity similar to Control_25 and Control_50 when mixed with PDMS. Even the same bioelastomers had different radical scavenging activity (%) between DPPH and ABTS assays. This is because the DPPH assay is suitable for measuring the antioxidant activity of lipophilic compounds, whereas the ABTS assay is appropriate for hydrophilic and lipophilic compounds [49]. In summary, as the powder content increased, both the content of phenolic compounds and the radical scavenging activity increased. A similar correlation between polyphenol and flavonoid content and antioxidant activity in plant ethanol extracts has been reported previously [50].

### 3.5. Antibacterial Activity of Bioelastomers

**Figure 5:**
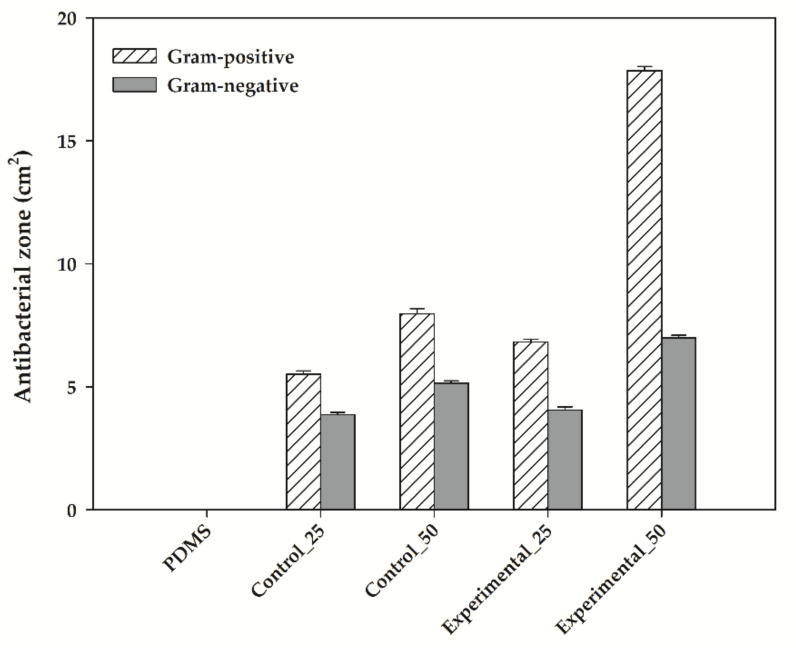
Antibacterial activities of the bioelastomers against Gram-positive (*S. aureus*) and Gram-negative (*E. coli*).

The presence of bacteria such as *S. aureus* and *E. coli* in food causes food spoilage, food-borne diseases, reduced shelf life, and economic loss [51]. Growth inhibition of these bacteria is considered an indicator of food safety [52]. Figure 5 demonstrates the antibacterial activity of the bioelastomers using agar plates for *S. aureus* and *E. coli*. In all agar plates with bioelastomers, bacteria around the bioelastomers were dead in antibacterial active zones. In order to measure the area of the antibacterial zones, the images were adjusted in contrast with a color threshold using Image J software. When comparing the effectiveness of the control and the experimental groups, the antibacterial zone by Experimental_50 was the greatest area among that of other bioelastomers (Figure 5). Porous morphology (Figure 2(e-2)) provided a relatively large surface area to release functional chemicals that kill bacteria [53]. Antibacterial activity of the bioelastomers significantly affected the Gram-positive bacterium *S. aureus*; however, the effect was not clearly observed for *E. coli*. Gram-positive bacteria do not have an outer membrane that protects cells from toxic molecules and provides an additional barrier to the inner membrane [54]. Catechins contained in aronia interact with the cell walls and membranes of bacterial cells and produce hydrogen peroxide, which causes fatal damage to bacterial cells [55]. Demirbas et al. [56] also demonstrated that the antibacterial activity of blackberry extract is only effective against Gram-positive *S. aureus*, not Gram-negative *E. coli*.

## 4. Conclusions

It was found that antioxidants remained in aronia juice processing residue, which was used as a raw material for the fabrication of functional bioelastomers. In bioelastomer fabrication, 25 and 50 wt% of the powder from aronia juice processing by-products were mixed in PDMS, and the powder of the freeze-dried aronia was used as a control group. In all experiments, the mechanical properties of the bioelastomer mixed with 50% powder were found to be unsuitable for use as a film-type of bioelastomer. As a result of surface morphology, it was found that the surface of the bioelastomer became rough when aronia powder was added, and the surface area increased as the content of aronia powder increased. However, compared to the PDMS film, there were no significant differences in the chemical properties of bioelastomers containing aronia powder. The antioxidant activity of the bioelastomer was approximately 1.4-fold lower than that of the control group; however, the antimicrobial activity was about 1.5-fold higher.

Through this study, we explored the possibility that food processing by-products could be used as functional materials, and their effect is expected to be significant if the mechanical properties of bioelastomers are improved. In the future, our research direction aims to improve the mechanical properties of bioelastomers and develop more economical and broader antimicrobial substances that can act on a variety of bacteria. Finally, this study, which directly utilized food processing by-products to produce functional materials, is expected to demonstrate an advantageous case for the development of economical biorefineries.

## Figures and Tables

**Table 1:** Summary of food processing by-products-based film-type functional materials: an overview of methods and emerging applications.

By-Products	Polymers	Methods	Applications	Ref.
Red beetroot (RB)	Polydimethylsiloxane (PDMS)	RB and corn starch were added to 14 mL of heptane	Food packaging	[13]
White and red grape seeds and, tomato skins and seeds	Polypropylene (PP)	Blending under a nitrogen stream at 180 °C, increasing the screw rate from 20 to 32 rpm for 10 min	Stabilization against thermal-oxidative degradation	[15]
Parsley and spinach stems, cocoa pod husks, and rice hulls	Microcrystalline cellulose (MCC)	Solution of 3% by weight of solids in Tetrahydrofuran for 29 days	Packaging and biomedicine	[16]
Chitin and chitosan from crab (*Carious mediterraneus*)	Polyvinyl alcohol (PVA)	Two grams of chitosan was dissolved in 100 mL of 2% (*v/v*) acetic acid at 25 °C for 24 h and 2 g of PVA was dissolved in 100 mL of distilled water at 80 °C for 6 h	Food packaging	[17]
Chardonnay grape pomace and turmeric waste (*Curcumina longa*)	Low-density polyethylene (LDPE)	Sample of 96/4 wt% LDPE/grape pomace waste, melt state at 140 °C with 160 rpm		[18]
Cocoa shell waste (CSW)	Polydimethylsiloxane (PDMS)	Micronized CSW was added to 10 mL of heptane	Food packaging and biomedical device	[19]
Carrot, radicchio, parsley, and cauliflower	Polyvinyl alcohol (PVA)	Powders were dispersed in 5% (*w/w*) water solution of HCl	Cosmetics and biodegradable polymer	[20]
Apricot (*Prunus armeniaca* L.) kernel skin (AKS)	Soy protein isolate (SPI)	SPI and glycerol were dispersed in 50 mL deionized water containing AKS at 80 °C for 30 min with 150 rpm	Food and drug packaging	[21]
Frozen blackberries (*Rubus fruticosus*)	Arrowroot starch	Arrowroot starch was dispersed in distilled water (4%, *w/w*) and mixed with glycerol and blackberry powder	Food packaging	[22]

**Table 2:** Experimental conditions for aronia bioelastomer formation.

Sample	Powder (g)	PDMS (g)	Powder (wt%)	PDMS (wt%)
PDMS	0	10	0	100
Control_25 ^1^	2.5	7.5	25	75
Control_50 ^1^	5.0	5.0	50	50
Experimental_25 ^2^	2.5	7.5	25	75
Experimental_50 ^2^	50	5.0	50	50

^1^ Bioelastomer with the freeze-dried aronia powder. ^2^ Bioelastomer with the aronia juice processing by-products.

**Table 3:** Total polyphenol and flavonoid contents of aronia powder and bioelastomers.

	Polyphenol Contents (mg GAE^1^/g Dry Powder)	Flavonoid Contents (mg RE^2^/g Dry Powder)
Aronia freeze-dried powder	42.0 ± 1.4	20.0 ± 1.1
Control_25	16.0 ± 0.5	8.0 ± 0.3
Control_50	18.0 ± 0.8	12.0 ± 0.4
Aronia juice processing by-products	37.2 ± 1.3	18.5 ± 0.8
Experimental_25	13.1 ± 0.3	4.4 ± 0.2
Experimental_50	15.3 ± 0.4	8.8 ± 0.3

^1^ GAE: gallic acid equivalent. ^2^ RE: rutin equivalent.

**Table 4:** Determination of radical scavenging activity of aronia powder and bioelastomers.

	Radical Scavenging Activity (%)	
	DPPH	ABTS
Aronia freeze-dried powder	89.4 ± 0.8	62.9 ± 2.6
Control_25	35.4 ± 0.1	16.7 ± 0.1
Control_50	49.6 ± 0.6	26.8 ± 1.1
Aronia juice processing by-products	63.8 ± 0.1	29.4 ± 0.3
Experimental_25	26.2 ± 0.2	12.4 ± 0.2
Experimental_50	35.7 ± 0.4	16.8 ± 0.1

## References

[1] Lee J.H., Yoo H.Y., Lee S.K., Chun Y., Kim H.R., Bankeeree W., Lotrakul P., Punnapayak H., Prasongsuk S., Kim S.W. (2020). Significant Impact of Casein Hydrolysate to Overcome the Low Consumption of Glycerol by *Klebsiella aerogenes* ATCC 29007 and Its Application to Bioethanol Production. Energy Convers. Manag..

[2] Kim H., Yoo H.Y., Kim Y.H., Kim I.K., Byun E.H., Yang Y.H., Park S.J., Na J.G., Shon H., Lee T. (2018). Improved Reutilization of Industrial Crude Lysine to 1,5-diaminopentane by Enzymatic Decarboxylation Using Various Detergents and Organic Solvents. Korean J. Chem. Eng..

[3] Huang T., Qian Y., Wei J., Zhou C. (2019). Polymeric Antimicrobial Food Packaging and Its Applications. Polymers.

[4] BCC Research Staff (2019). Sustainable Biopolymers: A BCC Research Outlook, BCC Research: Market Research Reports. https://www.bccresearch.com.

[5] Cinelli P., Coltelli M.B., Signori F., Morganti P., Lazzeri A. (2019). Cosmetic Packaging to Save the Environment: Future Perspectives. Cosmetics.

[6] Ul-Islam M., Ullah M.W., Khan S., Park J.K. (2020). Production of Bacterial Cellulose from Alternative Cheap and Waste Resources: A Step for Cost Reduction with Positive Environmental Aspects. Korean J. Chem. Eng..

[7] Narancic T., Cerrone F., Beagan N., O’Connor K.E. (2020). Recent Advances in Bioplastics: Application and Biodegradation. Polymers.

[8] Zerva I., Remmas N., Ntougias S. (2019). Biocatalyst Potential of Cellulose-Degrading Microorganisms Isolated from Orange Juice Processing Waste. Beverages.

[9] Lyu F., Luiz S.F., Azeredo D.R.P., Cruz A.G., Ajlouni S., Ranadheera C.S. (2020). Apple Pomace as a Functional and Healthy Ingredient in Food Products: A Review. Processes.

[10] Ben-Othman S., Jõudu I., Bhat R. (2020). Bioactives from Agri-Food Wastes: Present Insights and Future Challenges. Molecules.

[11] Seo H.S., Park B.H. (2020). Phenolic Compound Extraction from Spent Coffee Grounds for Antioxidant Recovery. Korean J. Chem. Eng..

[12] Takó M., Kerekes E.B., Zambrano C., Kotogán A., Papp T., Krisch J., Vágvölgyi C. (2020). Plant Phenolics and Phenolic-Enriched Extracts as Antimicrobial Agents against Food-Contaminating Microorganisms. Antioxidants.

[13] Tran T.N., Athanassiou A., Basit A., Bayer I.S. (2017). Starch-based Bio-elastomers Functionalized with Red Beetroot Natural Antioxidant. Food Chem..

[14] Quinto E.J., Caro I., Villalobos-Delgado L.H., Mateo J., de-Mateo-Silleras B., Redondo-Del-Río M.P. (2019). Food Safety through Natural Antimicrobials. Antibiotics.

[15] Cerruti P., Malinconico M., Rychly J., Matisova-Rychla L., Carfagna C. (2009). Effect of Natural Antioxidants on the Stability of Polypropylene Films. Polym. Degrad. Stab..

[16] Bayer I.S., Guzman-Puyol S., Heredia-Guerrero J.A., Ceseracciu L., Pignatelli F., Ruffilli R., Cingolani R., Athanassiou A. (2014). Direct Transformation of Edible Vegetable Waste into Bioplastics. Macromolecules.

[17] Hajji S., Chaker A., Jridi M., Maalej H., Jellouli K., Boufi S., Nasri M. (2016). Structural Analysis, and Antioxidant and Antibacterial Properties of Chitosan-poly(vinyl alcohol) Biodegradable Films. Environ. Sci. Pollut. Res. Int..

[18] Iyer K.A., Zhang L., Torkelson J.M. (2016). Direct Use of Natural Antioxidant-rich Agro-wastes as Thermal Stabilizer for Polymer: Processing and Recycling. ACS Sustain. Chem. Eng..

[19] Tran T.N., Heredia-Guerrero J.A., Mai B.T., Ceseracciu L., Marini L., Athanassiou A., Bayer I.S. (2017). Bioelastomers Based on Cocoa Shell Waste with Antioxidant Ability. Adv. Sustain. Syst..

[20] Perotto G., Ceseracciu L., Simonutti R., Paul U.C., Guzman-Puyol S., Tran T.N., Bayer I.S., Athanassiou A. (2018). Bioplastics from Vegetable Waste: Via an Eco-friendly Water-based Process. Green Chem..

[21] Li Y., Tang Z., Lu J., Cheng Y., Qian F., Zhai S., An Q., Wang H. (2019). The Fabrication of a Degradable Film with High Antimicrobial and Antioxidant Activities. Ind. Crops. Prod..

[22] Nogueira G.F., Fakhouri F.M., Velasco J.I., de Oliveira R.A. (2019). Active Edible Films Based on Arrowroot Starch with Microparticles of Blackberry Pulp Obtained by Freeze-Drying for Food Packaging. Polymers.

[23] Sidor A., Drożdżyńska A., Gramza-Michałowska A. (2019). Black Chokeberry (*Aronia melanocarpa*) and Its Products as Potential Health-promoting Factors—An overview. Trends Food Sci. Technol..

[24] Hidayat M.A., Maharani D.A., Purwanto D.A., Kuswandi B., Yuwono M. (2020). Simple and Sensitive Paper-based Colorimetric Biosensor for Determining Total Polyphenol Content of the Green Tea Beverages. Biotechnol. Bioprocess Eng..

[25] Oszmiański J., Lachowicz S. (2016). Effect of the Production of Dried Fruits and Juice from Chokeberry (*Aronia melanocarpa* L.) on the Content and Antioxidative Activity of Bioactive Compounds. Molecules.

[26] Mayer-Miebach E., Adamiuk M., Behsnilian D. (2012). Stability of Chokeberry Bioactive Polyphenols during Juice Processing and Stabilization of a Polyphenol-Rich Material from the By-Product. Agriculture.

[27] Dienaitė L., Pukalskas A., Pukalskienė M., Pereira C.V., Matias A.A., Venskutonis P.R. (2020). Phytochemical Composition, Antioxidant and Antiproliferative Activities of Defatted Sea Buckthorn (*Hippophaë rhamnoides* L.) Berry Pomace Fractions Consecutively Recovered by Pressurized Ethanol and Water. Antioxidants.

[28] Dilucia F., Lacivita V., Conte A., del Nobile M.A. (2020). Sustainable Use of Fruit and Vegetable By-Products to Enhance Food Packaging Performance. Foods.

[29] Choi M., Kang Y.R., Zu H.D., Lim I.S., Jung S.K., Chang Y.H. (2020). Effects of Time on Phenolics and in vitro Bioactivity in Autoclave Extraction of Graviola (*Annona muricata*) Leaf. Biotechnol. Bioprocess Eng..

[30] Chen Y.H., Yang C.Y. (2020). Ultrasound-Assisted Extraction of Bioactive Compounds and Antioxidant Capacity for the Valorization of *Elaeocarpus serratus* L. Leaves. Processes.

[31] Jayakumar A., Vedhaiyan R.K. (2020). Rapid Synthesis of Phytogenic Silver Nanoparticles Using *Clerodendrum splendens*: Its Antibacterial and Antioxidant Activities. Korean J. Chem. Eng..

[32] Kim H., Kim J.S., Kim Y., Jeong Y., Kim J.E., Paek N.S., Kang C.H. (2020). Antioxidant and Probiotic Properties of Lactobacilli and Bifidobacteria of Human Origins. Biotechnol. Bioprocess Eng..

[33] Shim S.E., Yashin V.V., Isayev A.I. (2004). Environmentally-friendly Physico-chemical Rapid Ultrasonic Recycling of Fumed Silica-filled Poly(dimethyl siloxane) Vulcanizate. Green Chem..

[34] Anatoly C., Pavel Z., Tatiana C., Alexei R., Svetlana Z. (2020). Water Vapor Permeability through Porous Polymeric Membranes with Various Hydrophilicity as Synthetic and Natural Barriers. Polymers.

[35] Gu Y., Qiu Y., Wei X., Li Z., Hu Z., Gu Y., Zhao Y., Wang Y., Yue T., Yuan Y. (2020). Characterization of Selenium-containing Polysaccharides Isolated from Selenium-enriched Tea and Its Bioactivities. Food Chem..

[36] Ceseracciu L., Heredia-Guerrero J.A., Dante S., Athanassiou A., Bayer I.S. (2015). Robust and Biodegradable Elastomers Based on Corn Starch and Polydimethylsiloxane (PDMS). ACS Appl. Mater. Interfaces.

[37] Esteves A.C.C., Brokken-Zijp J., Laven J., Huinink H.P., Reuvers N.J.W., van M.P., de With G. (2009). Influence of Cross-linker Concentration on the Cross-linking of PDMS and the Network Structures Formed. Polymer.

[38] Kurek M., Garofulić I.E., Bakić M.T., Ščetar M., Uzelac V.D., Galić K. (2018). Development and Evaluation of a Novel Antioxidant and pH Indicator Film Based on Chitosan and Food Waste Sources of Antioxidants. Food Hydrocoll..

[39] Bamba B.S.B., Shi J., Tranchant C.C., Xue S.J., Forney C.F., Lim L.T. (2018). Influence of Extraction Conditions on Ultrasound-Assisted Recovery of Bioactive Phenolics from Blueberry Pomace and Their Antioxidant Activity. Molecules.

[40] Zhu M., Huang Y., Wang Y., Shi T., Zhang L., Chen Y., Xie M. (2019). Comparison of (Poly)phenolic Compounds and Antioxidant Properties of Pomace Extracts from Kiwi and Grape Juice. Food Chem..

[41] Goldsmith C.D., Vuong Q.V., Stathopoulos C.E., Roach P.D., Scarlett C.J. (2018). Ultrasound Increases the Aqueous Extraction of Phenolic Compounds with High Antioxidant Activity from Olive Pomace. LWT.

[42] Lin M., Zhang J., Chen X. (2018). Bioactive Flavonoids in *Moringa oleifera* and their Health-promoting Properties. J. Funct. Foods.

[43] Ordóñez-Díaz J.L., Hervalejo A., Pereira-Caro G., Muñoz-Redondo J.M., Romero-Rodríguez E., Arenas-Arenas F.J., Moreno-Rojas J.M. (2020). Effect of Rootstock and Harvesting Period on the Bioactive Compounds and Antioxidant Activity of Two Orange Cultivars (‘Salustiana’ and ‘Sanguinelli’) Widely Used in Juice Industry. Processes.

[44] Sharma S., Barkauskaite S., Duffy B., Jaiswal A.K., Jaiswal S. (2020). Characterization and Antimicrobial Activity of Biodegradable Active Packaging Enriched with Clove and Thyme Essential Oil for Food Packaging Application. Foods.

[45] Hwang S.J., Yoon W.B., Lee O.H., Cha S.J., Kim J.D. (2014). Radical-scavenging-linked Antioxidant Activities of Extracts from Black Chokeberry and Blueberry Cultivated in Korea. Food Chem..

[46] Línzembold I., Czett D., Böddi K., Kurtán T., Király S.B., Gulyás-Fekete G., Takátsy A., Lóránd T., Deli J., Agócs A. (2020). Study on the Synthesis, Antioxidant Properties, and Self-Assembly of Carotenoid–Flavonoid Conjugates. Molecules.

[47] Ushasree M.V., Lee E.Y. (2020). Flavonoids, Terpenoids, and Polyketide Antibiotics: Role of Glycosylation and Biocatalytic Tactics in Engineering Glycosylation. Biotechnol. Adv..

[48] Park S.H., Kim J.C. (2019). Monoolein Cubosomes for Enhancement of in vitro Anti-oxidative Efficacy of Bambusae Caulis in Taeniam Extract Toward Carcinogenic Fine Dust-stimulated RAW 264.7 cells. Korean J. Chem. Eng..

[49] Kusumah J., Real Hernandez L.M., Gonzalez de Mejia E. (2020). Antioxidant Potential of Mung Bean (*Vigna radiata*) Albumin Peptides Produced by Enzymatic Hydrolysis Analyzed by Biochemical and In Silico Methods. Foods.

[50] Wan Yahaya W.A., Abu Yazid N., Mohd Azman N.A., Almajano M.P. (2019). Antioxidant Activities and Total Phenolic Content of Malaysian Herbs as Components of Active Packaging Film in Beef Patties. Antioxidants.

[51] Xiao X.N., Wang F., Yuan Y.T., Liu J., Liu Y.Z., Yi X. (2019). Antibacterial Activity and Mode of Action of Dihydromyricetin from *Ampelopsis grossedentata* Leaves against Food-Borne Bacteria. Molecules.

[52] Liang S., Wang L. (2018). A Natural Antibacterial-Antioxidant Film from Soy Protein Isolate Incorporated with Cortex Phellodendron Extract. Polymers.

[53] Tsai Y.H., Yang Y.N., Ho Y.C., Tsai M.L., Mi F.L. (2018). Drug Release and Antioxidant/antibacterial Activities of Silymarin-zein Nanoparticle/bacterial Cellulose Nanofiber Composite Films. Carbohydr. Polym..

[54] Silhavy T.J., Kahne D., Walker S. (2010). The Bacterial Cell Envelope. Cold Spring Harb. Perspect. Biol..

[55] Nakayama M., Shimatani K., Ozawa T., Shigemune N., Tsugukuni T., Tomiyama D., Kurahachi M., Nonaka A., Miyamoto T. (2013). A Study of the Antibacterial Mechanism of Catechins: Isolation and Identification of *Escherichia coli* Cell Surface Proteins that Interact with Epigallocatechin Gallate. Food Control.

[56] Demirbas A., Yilmaz V., Ildiz N., Baldemir A., Ocsoy I. (2017). Anthocyanins-rich Berry Extracts Directed Formation of Ag NPs with the Investigation of their Antioxidant and Antimicrobial Activities. J. Mol. Liq..

